# Identification of unique species in the marine ecosystem based on the weighted trophic field overlap

**DOI:** 10.1016/j.mex.2019.02.024

**Published:** 2019-02-28

**Authors:** Zitao Xiao, Jiaying Wu, Binduo Xu, Chongliang Zhang, Yiping Ren, Ying Xue

**Affiliations:** a Fisheries College, Ocean University of China, Qingdao, 266003, China; b Laboratory for Marine Fisheries Science and Food Production Processes, Pilot National Laboratory for Marine Science and Technology (Qingdao), Qingdao, 266237, China; c CAS Key Laboratory of Marine Ecology and Environmental Sciences, Institute of Oceanology, Chinese Academy of Sciences, Qingdao, 266071, China

**Keywords:** Weighted trophic field overlap (WTO), Keystone species, Topological network, Interaction strength, Prey proportion, Food web

## Abstract

Based on the trophic field overlap of species in the food webs, we propose using the weighted trophic field overlap (WTO) to determine the uniqueness of species in a topological network by considering the food web structure and the proportions of prey in the diets of predators. This proposed method measures uniqueness structurally and mathematically and considers cannibalism and mutual predation between species to overcome the deficiencies of the traditional method (the sum of trophic field overlap, STO), which only relies on the topological structure of the food web. Species with the lowest WTO values have high interaction strengths with other species in the food web weighted by the proportion of prey and play important roles as prey in the initial ecosystem, which are not recognized by the traditional method. The proposed index is sensitive to changes in the diets of predators since slight fluctuations may cause the index to vary considerably. The proposed methodology could be extended to other marine ecosystems to identify unique species from a practical and dynamic perspective and will contribute to the protection of unique species that maintain the trophic diversity of food webs and ecosystem robustness.

• A WTO index was proposed for identifying unique species in food webs.
• This index considers both the topological network structure and proportion of prey.
• Cannibalism and mutual predation between species are also accounted for.

## Graphical abstract

**Figure:**
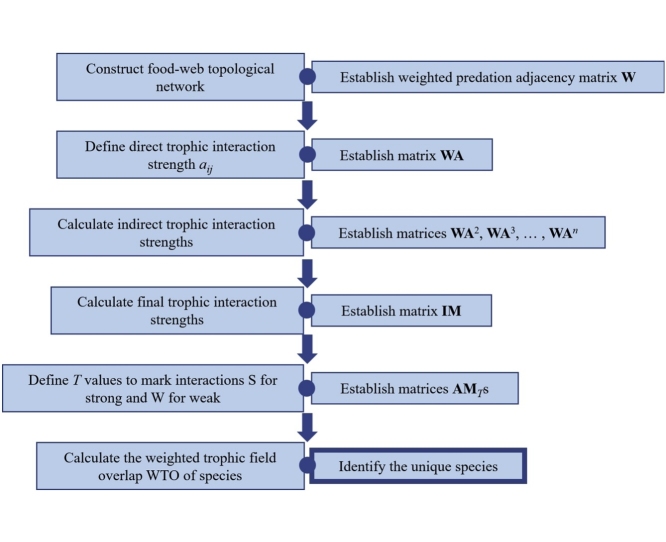

**Specifications Table**

**Table:** 

**Subject Area:**	Environmental Science
**More specific subject area:**	Marine Ecosystems and trophic networks
**Method name:**	Weighted trophic field overlap (WTO)
**Name and reference of original method**	Lai, S., et al., 2015. A trophic overlap-based measure for species uniqueness in ecological networks. Ecol. Modell. 299, 95-101.
**Resource availability**	–

## Method details

The uniqueness of a species is based on its irreplaceability in the food web and is considered when identifying important species [[1], [2], [3], [4]]. Species with unique topological positions and trophic interactions [[1], [2], [3]] that overlap less with those of other species can help maintain high trophic diversity in an ecosystem [1]. Thus, uniquely important species should be protected.

Mathematical methods and the topological network perspective [5] are required to quantify the interactions among species. The topological methodology (the sum of trophic field overlap, STO) proposed by Lai et al. [6] provides an alternative means of identifying the unique species with the lowest trophic field overlap. This method elucidates the structural uniqueness of a certain species, however, it over-depends on the topological network structure and neglects the quantitative effects of predation, which affect the accurate identification of unique species in the food web. Link weight in the food-web network means the relative importance of links and the strength of trophic interaction [[7], [8], [9]]. Therefore, prey proportion as link weight in the food web should considered. Besides, the STO index does not mention cannibalism and mutual predation that can be observed commonly in the marine ecosystem. They can affect species directly and indirectly [10,11] and drive the dynamics of populations or structures [[12], [13], [14]]. Therefore, cannibalism and mutual predation also should be considered when quantifying the interaction strength among species.

In this paper, we measured the trophic field overlap of species by considering a weighted topological network both structurally and mathematically. Cannibalism and mutual predation between species were accounted for in the calculations. Regression and non-parametric test were used for comparison. This method reflects the irreplaceability of species by considering the weighted trophic field overlap (WTO) and overcomes the drawbacks of the traditional method (STO). Thus, it will contribute to the protection of unique species and maintenance of trophic diversity and ecosystem robustness. In the following sections, we will (1) state the calculation process of the STO in brief and WTO in detail; (2) state the shortcomings of the method and future research directions; (3) compare results between indicators (WTO and STO, as well as them and other topological indicators respectively), and discuss the ecological meanings of WTO and unique species based on correlation and ecological background by case study.

### Uniqueness according to the STO method of Lai et al. [6]

Firstly, the direct interaction strength of species *i* on another species *j* was quantified by the reciprocal of the *D* value of *j* (i.e., the number of nodes directly connected to *j*) if there was direct predation between them. The indirect interaction strengths were calculated based on the direct interactions as described by Lai et al. [6]. Secondly, the maximum shortest length of the food-web network plus 1 (i.e. *n*) dividing the sum of the direct and indirect interactions of species *i* on other species was defined as the final interaction strength of a species *i*. The threshold variable *T*, which increases from 0 to 1 in increments of 0.001, was used to identify strong (S) and weak (W) interactions. The value of a for species *i* was designated as S when *a* > *T* and W when *a* ≤ *T*. Thirdly, the STOs of all species were counted across the entire range of *T* values.

### Uniqueness according to WTO

Each link of the nodes was weighted by the weight percentage of each prey to illustrate the relative importance of links in the food-web network. The adjacency matrix of all species was constructed based upon the trophic relationships in the food web. We replaced all of the values in the matrix with the weight percentages of prey. Then, the sum of the weight percentages in each row was defined as the weighted out-degree *WD_out_*, and the sum of those in each column was designated as the weighted in-degree. The sum of the row and column values for species *i* was defined as the weighted degree *WD_i_* of species *i*. For the weighted adjacency matrix **W**, the sum of each column (i.e., weighted in-degree) equals 1, while the row sums (i.e., *WD_out_*) corresponding to the outgoing effects of species are not equal. The latter feature reflects the importance of species as prey in the whole food web and plays a significant role in calculating interaction strength.

The direct interaction strength of species *i* on *j* can be weaker if the latter has more neighbors [15]. Lai et al. [6] quantified the direct interaction strength by defining it as the reciprocal of *D*, which was defined as the reciprocal of the weighted degree in the improved method. Thus, the direct interaction strength of species *i* on *j* (*a_ij_*) is defined as

(1) $$a_{ij}=\frac{W_{ij}}{WD_{j}},$$

where *W_ij_* represents the weight proportion of *i* in the total intake of *j* or *j* in the total intake of *i* in the food web, and *WD_j_* represents the weighted degree of species *j*. Note that if there is no predation between *i* and *j*, *a_ij_* = 0. When all one-step effects (i.e., direct interactions) between species are calculated, a matrix **WA** can be constructed, where the *ij*th element *a_ij_* represents the direct interaction strength of species *i* on *j*. Here, *i* corresponds to the row number and *j* corresponds to the column number.

To calculate the weighted degree, the directed network including loop chains with single-node (cannibalism) and dual-direction links (mutual predation between two species) needs to be transformed into undirected network. The prey proportions attached to loop chains were retained the same as one-way links, while the pairs of those from mutual predation between two species were added up.

Indirect interaction is defined as a trophic relationship between species that interact through one or more than one intermediate species [5], and it can be quantified by using direct interactions. In a two-step food-web network, the strength of the two-step interaction is the product of the strengths of the two direct interactions via the matrix power operation **WA**^2^. For example, if species *i* has a connection with *j* through species *k* (i.e., *i* can interact with *j* in two steps and the length of the links between the two nodes is 2), then probability theory makes it possible to quantify the two-step interaction (namely, *a_ikj_*) of species *i* on *j* as follows

(2) $$a_{ikj}=a_{ik}\times a_{kj},$$

where *a_ikj_* represents the two-step interaction of species *i* on *j* through species *k*, and *a_ik_* and *a_kj_* represent the one-step interactions of species *i* on *k* and *k* on *j*, respectively. Thus, all of the possible two-step interactions between species *i* and *j* from various paths through other intermediate species can be calculated as

(3) $$sa_{ij}=a_{i1}\times a_{1j}+a_{i2}\times a_{2j}+a_{i3}\times a_{3j}+\cdots +a_{iN}\times a_{Nj},$$

where *sa_ij_* represents the two-step interaction of species *i* on *j* in the food network, and *N* represents the number of species in the food web. The operational rule according to Eq. (3) conforms to the matrix power operation algorithm; thus, all two-step interactions can be computed as **WA**^2^. The interaction strengths (in matrix **IM**) between pairwise species for the *n*-step food-web network are the mean values of all interaction matrices

(4) $$IM=\frac{1}{n}\left(WA+WA^{2}+WA^{3}+\cdots +WA^{n}\right),$$

where the *ij*th element *IM_ij_* in matrix **IM** represents the interaction strength between species *i* and *j* in the food web, and *n* represents the maximum shortest distance in the food network plus 1 (the step length used in the analysis should be slightly greater than the maximum shortest distance to ensure that the effect of one species can spread to all other parts of the food web; see Lai et al. [6]).

The threshold variable T, which increases from 0 to 1 in increments of 0.00001, was used to identify strong (S) and weak (W) interactions. All of the interactions *a_ij_* were marked S when *a* > *T* and W when *a* ≤ *T* under different *T* values, and a series of matrices **AM***_T_* was constructed. As mentioned previously, trophic field overlap describes the similarity of the interactions among all species in the food web. Note that the W trophic interactions are responsible for food web stability [16] and that they are as important as S interactions [6]. The number of instances in which the marks in row *i* and the other rows are the same, column by column, is counted as the weighted trophic field overlap (WTO) of *i* and other species under a given *T*. For example, for column *j* and a given threshold *T_1_*, the WTO*_ijk_*,*_T1_* (WTO of *i* and *k* for *j* under *T_1_*) can be counted as 1 when *AM_ij_* in row *i* and *AM_kj_* in row *k* are both S or both W. This process can be repeated for all of the columns to obtain the WTO*_ik_*,*_T1_* (WTO of *i* and *k* under *T_1_*, which equals the difference between N and the Hamming Distance of row *i* and *k* in matrix **AM***_T1_*) and repeated for all rows with row *i* to obtain the WTO*_i_*,*_T1_* (WTO of *i* under *T_1_*, which equals the sum of the differences between N and the Hamming Distances of row *i* and other rows in matrix **AM***_T1_*). After counting in every matrix **AM***_T_* across the entire range of *T* values, add up all WTOs and the result is the new uniqueness value of *i*. Small WTO values indicate low trophic field overlap of one species with all others, and less overlapped species are considered unique.

All of the analyses were conducted using the Ucinet 6 and Matlab r2006a software.

### The shortcomings and future study

Both the network structure and weight percentage of prey could be affected by the taxonomic category. The lower the taxonomic category chosen, the more accurate the results. Species with relatively high weight percentages in diets tend to play more important roles and have stronger interactions with other species. However, the index of weight percentage could overestimate the importance of species with high body masses or sizes [4,7]. In the future, efforts should be made to examine the diet matrix with additional objective and comparable indices, such as the importance index. Meanwhile, the identification of unique species could be improved by taking into account additional factors. Moreover, the interaction strength can be further classified into different levels, such as strong, intermediate, and weak.

This index is sensitive to changes in the diets of predators since slight fluctuations may cause it to vary considerably. In a dynamic ecosystem, the diet compositions and feeding intensities of species vary considerably and may change the strengths of the trophic interactions among species and influence the uniqueness of species in the food web; thus, they should be considered for accurate identification of unique species. Moreover, the external disturbances to ecosystems affect the trophic interaction structure and strength directly or indirectly, and the net production and internal structure will change when the ecosystem gets mature. These will also change the relative uniqueness of species and the mechanisms are complex. Thus, these need to be studied further to determine how disturbances and maturity of ecosystem will affect species uniqueness in the food web.

### Case study and ecological meanings

**Fig. 1:**
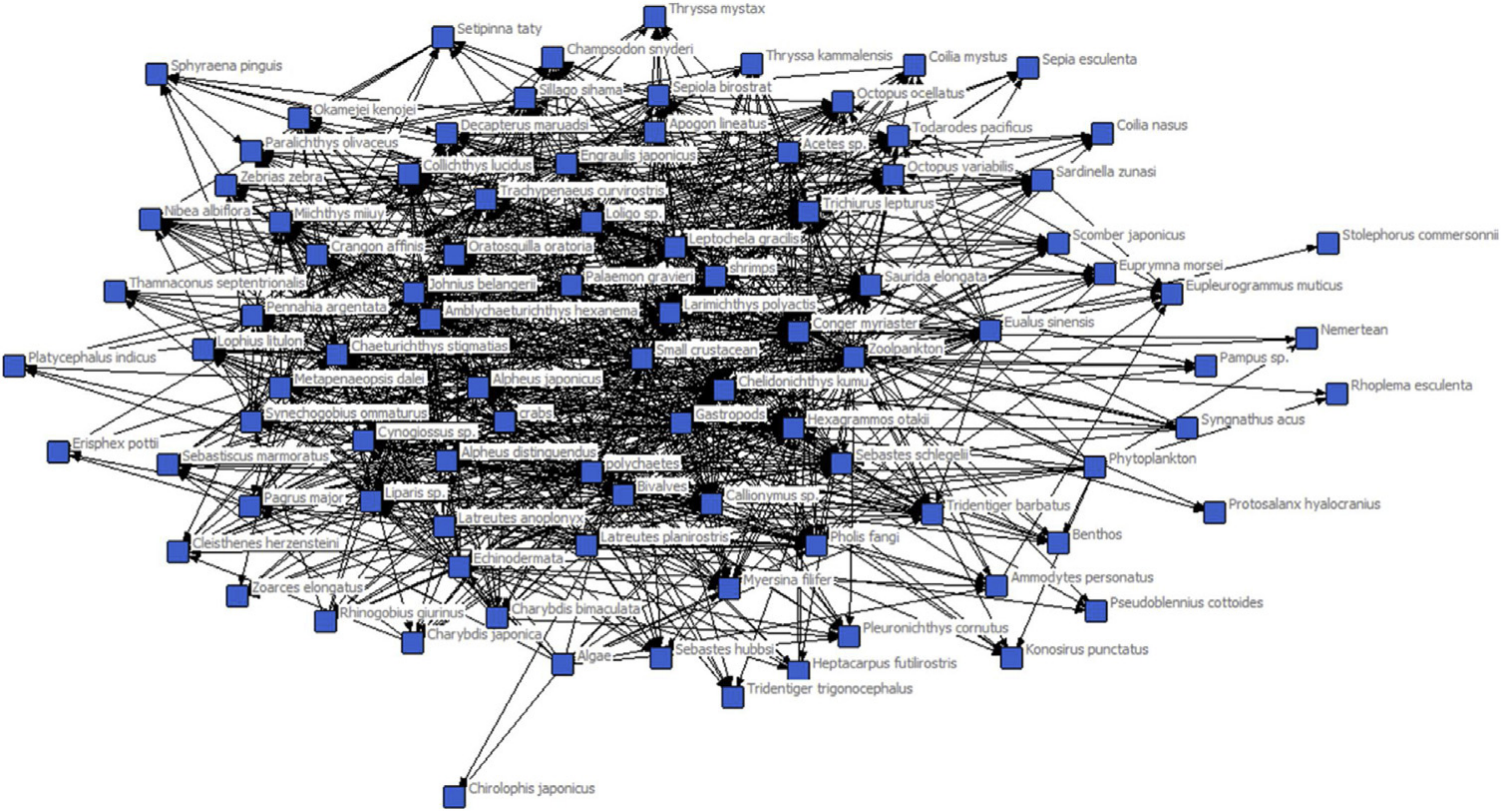
Food web diagram representing trophic interactions among species in the Haizhou Bay food web. Each node represents either a species or a trophspecies. The links connecting the nodes stand for trophic interactions (each arrow leaves the prey and enters the predator).

An ecological network (Fig. 1) was constructed based on data collected from five bottom trawl surveys in Haizhou Bay that were performed during March, May, July, September, and December of 2011 (Further details are available in the literature [[17], [18], [19]]). There were 93 nodes representing each species or aggregated trophic group (Table 1) and 2042 links representing the trophic flows between these trophic groups.

**Table 1:** Species analyzed in the Haizhou Bay food web.

Nodes	Species	Nodes	Species	Nodes	Species	Nodes	Species
1	*Eualus sinensis*	25	*Rhoplema esculenta*	49	Nemertean	73	*Apogon lineatus*
2	*Pennahia argentata*	26	*Latreutes anoplonyx*	50	*Johnius belangerii*	74	*Alpheus distinguendus*
3	*Konosirus punctatus*	27	*Sebastiscus marmoratus*	51	*Rhinogobius giurinus*	75	*Callionymus* sp.
4	*Pampus* sp.	28	*Paralichthys olivaceus*	52	*Heptacarpus futilirostris*	76	*Eupleurogrammus muticus*
5	*Thryssa kammalensis*	29	polychaetes	53	*Synechogobius ommaturus*	77	*Larimichthys polyactis*
6	*Hexagrammos otakii*	30	*Lophius litulon*	54	shrimps	78	*Chelidonichthys kumu*
7	*Protosalanx hyalocranius*	31	*Nibea albiflora*	55	*Loligo* sp.	79	crabs
8	*Zebrias zebra*	32	*Setipinna taty*	56	*Sardinella zunasi*	80	*Conger myriaster*
9	*Trichiurus lepturus*	33	Echinodermata	57	*Alpheus japonicus*	81	*Sebastes schlegelii*
10	*Metapenaeopsis dalei*	34	*Crangon affinis*	58	*Scomber japonicus*	82	*Trachypenaeus curvirostris*
11	*Coilia nasus*	35	Small crustacean	59	*Charybdis japonica*	83	*Platycephalus indicus*
12	Benthos	36	*Stolephorus commersonnii*	60	*Cynogiossus* sp.	84	*Sphyraena pinguis*
13	*Pagrus major*	37	*Pleuronichthys cornutus*	61	*Liparis* sp.	85	*Latreutes planirostris*
14	*Pseudoblennius cottoides*	38	*Sepia esculenta*	62	*Charybdis bimaculata*	86	*Ammodytes personatus*
15	*Champsodon snyderi*	39	*Sebastes hubbsi*	63	*Sepiola birostrat*	87	*Palaemon gravieri*
16	*Octopus ocellatus*	40	*Okamejei kenojei*	64	Bivalves	88	*Zoarces elongatus*
17	*Pholis fangi*	41	*Oratosquilla oratoria*	65	*Euprymna morsei*	89	*Octopus variabilis*
18	*Coilia mystus*	42	*Decapterus maruadsi*	66	*Chirolophis japonicus*	90	*Saurida elongata*
19	Zoolpankton	43	*Amblychaeturichthys hexanema*	67	*Todarodes pacificus*	91	*Myersina filifer*
20	Phytoplankton	44	*Acetes* sp.	68	*Engraulis japonicus*	92	*Thryssa mystax*
21	Gastropods	45	*Chaeturichthys stigmatias*	69	*Thamnaconus septentrionalis*	93	*Tridentiger barbatus*
22	*Cleisthenes herzensteini*	46	*Collichthys lucidus*	70	*Tridentiger trigonocephalus*		
23	*Syngnathus acus*	47	*Erisphex pottii*	71	*Sillago sihama*		
24	Algae	48	*Miichthys miiuy*	72	*Leptochela gracilis*		

By considering the effects up to eight steps (*n* = 8), *T* varied from 0 to 1 in increments of 0.001 and 0.00001, and STO and WTO values were calculated for individual species in the Haizhou Bay food web. The species corresponding to nodes 72, 55, 57, 43, and 77 (i.e., *Leptochela gracilis*, *Loligo* sp., *Alpheus japonicus*, *Amblychaeturichthys hexanema*, and *Larimichthys polyactis*) were the most unique species with the lowest STOs. The species corresponding to nodes 72, 44, 68, 57, and 74 (i.e., *Leptochela gracilis*, *Acetes* sp., *Engraulis japonicus*, *Alpheus japonicus*, and *Alpheus distinguendus*) were the most unique species with the lowest WTOs (Table 2).

**Table 2:** Species ranks according to the two uniqueness measures for the Haizhou Bay food web.

Ranks	STO	WTO	Ranks	STO	WTO	Ranks	STO	WTO	Ranks	STO	WTO
1	72	72	21	38	83	41	92	31	61	28	86
2	55	44	22	11	25	42	18	88	62	63	51
3	57	68	23	34	47	43	69	37	63	30	32
4	43	57	24	9	17	44	58	7	64	31	16
5	77	74	25	70	36	45	60	48	65	40	65
6	41	85	26	83	45	46	73	59	66	62	89
7	36	34	27	3	90	47	1	92	67	48	78
8	80	55	28	87	42	48	26	38	68	15	22
9	74	10	29	68	75	49	17	93	69	67	46
10	45	41	30	84	84	50	37	28	70	8	60
11	66	73	31	76	82	51	46	81	71	27	27
12	7	77	32	85	23	52	39	40	72	42	70
13	6	43	33	50	50	53	10	62	73	5	18
14	25	87	34	86	30	54	75	52	74	81	6
15	14	26	35	23	3	55	2	91	75	71	13
16	78	14	36	65	56	56	56	58	76	16	80
17	44	66	37	52	76	57	51	39	77	89	15
18	4	9	38	88	1	58	22	4	78	91	63
19	47	5	39	32	67	59	61	61	79	93	8
20	82	11	40	90	69	60	59	2	80	13	71

*Note*: STO and WTO are the sums of all trophic field overlap derived from Lai et al. [6] and the improved method, respectively. Numbers in columns named STO and WTO represent the nodes of species in Table 1.

**Fig. 2:**
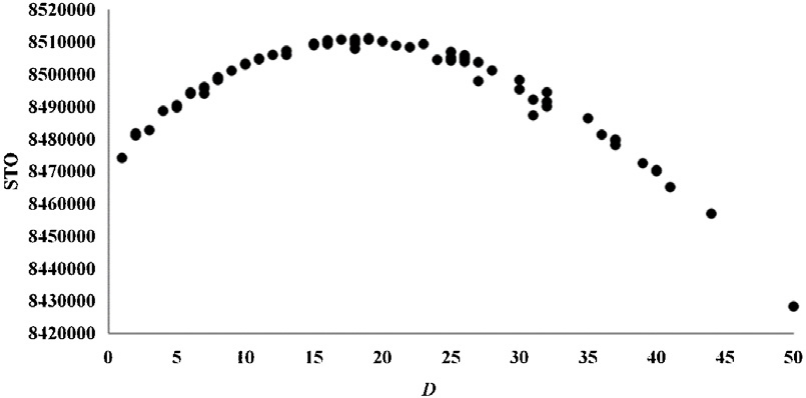
STO versus *D* according to the uniqueness measure (in a binary network) proposed by Lai et al. [6] for all species in the Haizhou Bay ecosystem.

**Fig. 3:**
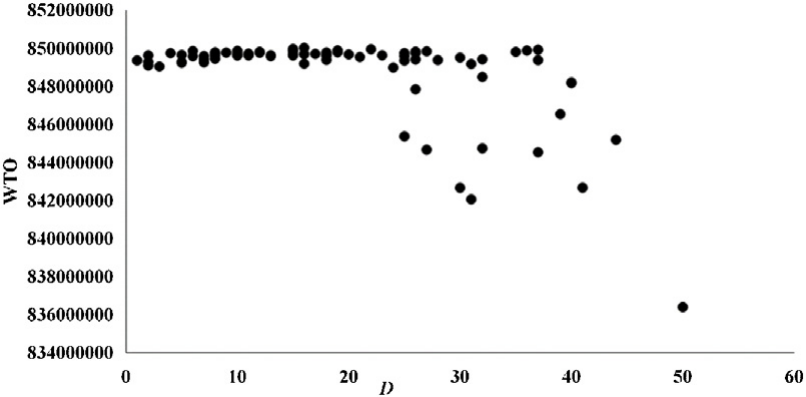
WTO versus *D* according to the improved method (in a weighted network) proposed in this study for all species in the Haizhou Bay ecosystem.

**Fig. 4:**
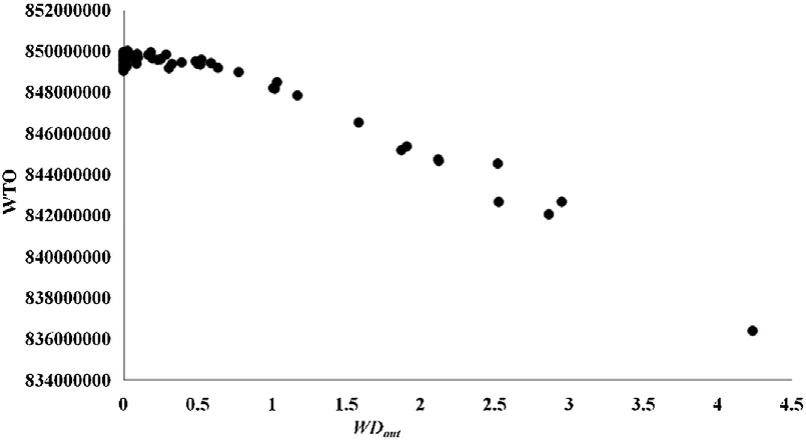
WTO versus *WD_out_* according to the improved method (in a weighted network) proposed here for all species in the Haizhou Bay ecosystem.

The results of species ranking according to WTO and STO were significantly different (*P* < 0.01) using the Wilcoxon signed-rank test. Significant negative correlations were detected between WTO and three topological indices, i.e., degree *D*, *WD_out_*, and the bottom-up keystone index *K_b_* [5] (Table 3) using Spearman rank correlation. STO was not significantly related to these quantities. The analyses were conducted in SPSS 21.0. *D* had a clear relationship with the STO according to the curve estimation (Fig. 2), reflecting the STO depends only on the topological structure. There was no correlation between WTO and *D* (Fig. 3), so the uniqueness values calculated using the WTO method did not depend only on the topological structure. The negative correlation between WTO and *WD_out_* (Fig. 4) suggests that species uniqueness has a positive correlation with *WD_out_*. Generalist predators with high *WD_out_* tend to have high uniqueness, and those with low *WD_out_* tend to have low uniqueness. Apex predators with *WD_out_* = 0 have a high range of WTO values and tend to have relatively low uniqueness. These results demonstrate the importance of unique species with lowest WTO as prey in Haizhou Bay ecosystem, since *WD_out_* is helpful for finding forage species and *K_b_* reflects the bottom-up effect [20].

**Table 3:** Spearman rank correlation coefficients between STO and WTO and three topological indices.

	*D*	*WD_out_*	*K_b_*
STO	−0.120	−0.168	−0.135
WTO	*−0.276*	**−0.545**	**−0.445**

*Note*: Bold values indicate significant correlations at the 0.01 level, the italic value indicates significance at the 0.05 level, and the values appearing in normal font indicate non-significant correlations.

Unique species exist in every food web. They hold important positions in the food web and act as connections between species with their unique positions and trophic interactions, making the web even closer and reducing the loss of energy transmitted through the food chain. Moreover, unique species with the lowest WTO were found to have higher interaction strengths with other species in matrix **IM**. The interaction strength distribution showed that there were many weak interactions and few strong interactions in the food web [15,21]. Thus, strong interactors tend to be unique and irreplaceable due to their distinctive trophic interactions, which contribute to the trophic diversity and ecosystem robustness. Unique species play important roles as essential prey and have uniquely strong interaction strength with other species in the food web, given the current ecosystem configuration. The roles of unique species may differ in different ecosystems since other configurations may also provide ecosystem services, but their uniquely strong trophic interaction strengths still ensure their important positions in the food web. This is determined by the algorithm of WTO and the characteristics of ecosystem with many weak interactions and few strong interactions.
